# The effect of implementing CT‐scan guidelines in the management of pediatric trauma: Protocol for a scoping review

**DOI:** 10.1111/aas.70024

**Published:** 2025-03-18

**Authors:** Andreas R. Slente, Emma A. Tsuchiya, Mikkel H. Jensen, Emilie B. Øberg, Johan Heiberg

**Affiliations:** ^1^ Department of Anaesthesiology Center of Head and Orthopedics, Rigshospitalet Copenhagen Denmark; ^2^ Department of Clinical Medicine University of Copenhagen Copenhagen Denmark

**Keywords:** child, computed tomography, imaging guidelines, pediatrics, protocol, scoping review, trauma

## Abstract

**Background:**

Trauma is a leading cause of death and disability in the pediatric population. CT has gained a key role in the management of pediatric trauma. Recently, there has been an increased focus on limiting exposure to ionizing radiation in pediatric patients. Furthermore, most CT scans performed in the management of pediatric trauma are without clinically relevant findings. Therefore, the proposed scoping review aims to assess the effects of introducing a guideline addressing whether pediatric trauma patients should undergo a CT scan.

**Methods:**

In accordance with the Preferred reporting items for systematic review and meta‐analysis extension for scoping reviews (PRISMA‐ScR) we will conduct a scoping review assessing the effect of implementing CT‐scan guidelines in the management of pediatric trauma. We will examine study design, patient characteristics, details of implemented guidelines, and outcomes across included studies.

**Results:**

Results will be reported both descriptively and in tabulated form.

**Conclusion:**

The proposed scoping review will provide an overview of the published evidence concerning the introduction of CT‐scan guidelines for the management of pediatric trauma. This might aid in the implementation of further initiatives to reduce ionizing radiation exposure in the pediatric trauma population.

## 1 BACKGROUND

Traumatic injuries are a leading cause of death in the pediatric population.^1^, ^2^, ^3^ The initial evaluation of pediatric patients presenting with trauma is complicated by the inherent challenges in collecting a pediatric history. These include the patient's inability to precisely provide anamnestic information, emotional distress in the child, decreased levels of consciousness, and so forth. Computerized tomography (CT) has gained a key role in the management of pediatric trauma, with studies citing that over half of presenting pediatric trauma patients receive a CT scan during initial evaluation.^4^, ^5^, ^6^

The pediatric population is more sensitive to the carcinogenic consequences of ionizing radiation compared to the adult population.^7^, ^8^ It has been proposed that this is due to the greater proportion of dividing cells in children and their longer life expectancy after exposure, in which a cancer might develop.^8^ Miglioretti et al. projected that 1 in every 300 to 390 abdomen/pelvis CT scans performed on girls would result in a radiation‐induced solid cancer.^9^

Due to its carcinogenic effect, there has been an increased focus on reducing ionizing radiation in the pediatric population. Furthermore, the great majority of performed CT scans of pediatric trauma patients are without clinically significant findings.^4^, ^6^ Thereby, there is both an incentive and a potential to decrease the number of CT scans in the management of pediatric trauma. Therefore, this scoping review aims to assess the effects of introducing a guideline addressing whether pediatric trauma patients should undergo a CT scan.

## 2 METHODS

This protocol has been written in accordance with the preferred reporting items for systematic review and meta‐analysis protocols (PRISMA‐P) 2015 statement.^10^ The scoping review following this protocol will be written in accordance with the preferred reporting items for systematic review and meta‐analysis extension for scoping reviews (PRISMA‐ScR).^11^

### 2.1 Research questions

- Does the introduction of a guideline or protocol addressing whether a CT scan should be performed in pediatric trauma patients affect the proportion of patients undergoing a CT scan at initial evaluation?
- What characterizes the guidelines or protocols used?
- What desirable and undesirable outcomes have been assessed?

### 2.2 Types of studies

We will include studies from 2010 and onwards, evaluating the effect of implementing a guideline or protocol addressing whether a CT scan should be performed in a pediatric trauma population. Studies will be included regardless of language or publication source.

### 2.3 Types of participants

Studies evaluating the treatment of pediatric trauma patients (age <18) presenting to a trauma center or emergency room will be included.

### 2.4 Intervention and comparator

The intervention in the included studies will be the implementation of a guideline or protocol addressing whether the CT scan should be performed. The included studies must include data concerning the frequency of CT scans after guideline implementation, compared to data from the same center prior to the implementation of the guideline or protocol.

### 2.5 Types of outcome measures

All reported outcomes will be conveyed.

### 2.6 Electronic searches

We will systematically search the following databases for relevant literature: Medline, EMBASE, and Web of Science. A preliminary search history in Medline will be available in the Appendix. We will conduct a search for any ongoing trials on clinicaltrials.gov. If any new and relevant search terms arise, they will be included in a new search history. An updated search history will be available in the Appendix of the review, if relevant. Suitable articles and reviews will also be manually searched for literature meeting inclusion criteria.

### 2.7 Data collection and analysis

#### 2.7.1 Selection of studies

Using Covidence, two reviewers will independently screen the titles and abstracts of studies found in the systematic search. Studies that have not been excluded during screening will then be assessed in full text for inclusion. Any conflicts in screening and assessment of full text eligibility will be resolved by reaching a consensus or by discussion with a third reviewer. The selection process will be conveyed in the final review via a PRISMA flowchart.^12^

#### 2.7.2 Data extraction and management

The reviewer will develop a predefined data extraction form for independent data extraction. The extracted data will include study characteristics (year of publication, study design, study duration, country), participants (number of participants, type of trauma, sex, and age), intervention (characteristics of guideline) and all patient‐relevant and non‐patient‐relevant outcomes.

#### 2.7.3 Strategy for data synthesis

Data will be reported both descriptively and in tabulated form.

## 3 DISCUSSION

The described review will shed light on the existing evidence of the effect of implementing a guideline addressing which patients should receive a CT scan in the management of pediatric trauma. Due to the increased attention on decreasing exposure to ionizing radiation in the pediatric population, knowledge on the impact of introducing CT scan reducing guidelines might aid in their implementation. Strengths of this review are the predefined protocol and systematic literature search in accordance with PRISMA statements.^10^, ^11^, ^12^

The proposed review also has some limitations. These include an expected heterogeneity in implemented guidelines and assessed outcomes. Furthermore, we will not examine each included study for risk of bias.

## 4 CONCLUSION

The presented review will give an overview of the available evidence on implementing guidelines addressing which patients should undergo CT scans in the management of pediatric trauma. This might aid in the implementation of further initiatives to decrease exposure to ionizing radiation in children presenting with trauma.

## AUTHOR CONTRIBUTIONS

Andreas R. Slente drafted the manuscript for this protocol. Emma A. Tsuchiya, Mikkel H. Jensen, Emilie B. Øberg, and Johan Heiberg contributed to the conceptualization and drafting process. Andreas R. Slente and Emma A. Tsuchiya developed the search strategy. All authors have reviewed the manuscript, had the opportunity to provide feedback, and approved the final draft of this protocol.

## FUNDING INFORMATION

Andreas R. Slente did not receive any funding for the work in this protocol, or in the following review. No funder, sponsor, department, or institution had any influence on the writing of this protocol or review.

## Data Availability

Data sharing not applicable—no new data generated.

## OVID MEDLINE(R) ALL <1946 TO FEBRUARY 14, 2025>

**Table:**

1.	exp Tomography, X‐Ray Computed/	518,003
2.	((CT or cross‐sectional or CAT) adj3 (scan* or imag*)).mp.	210,324
3.	1 or 2	616,726
4.	exp Clinical Protocols/	199,082
5.	practice guideline/	33,080
6.	(Protocol* or guideline* or prediction rule*).mp.	1,459,609
7.	4 or 5 or 6	1,459,646
8.	exp Child/	2,251,587
9.	exp Pediatrics/	64,856
10.	Adolescent/	2,306,869
11.	(Child* or pediat* or paediat* or infant* or adolescen* or juvenile* or toddler* or neonate*).mp.	4,923,255
12.	8 or 9 or 10 or 11	4,924,448
13.	exp Wounds/ and Injuries/	83,640
14.	(Injur* or traum*).mp.	1,764,911
15.	13 or 14	1,764,911
16.	3 and 7 and 12 and 15	1429
